# Predictive saccades in children and adults: A combined fMRI and eye tracking study

**DOI:** 10.1371/journal.pone.0196000

**Published:** 2018-05-02

**Authors:** Katerina Lukasova, Mariana P. Nucci, Raymundo Machado de Azevedo Neto, Gilson Vieira, João R. Sato, Edson Amaro

**Affiliations:** 1 LIM-44, NIF - Neuroimagem Funcional, Faculdade de Medicina, Universidade de São Paulo (USP), São Paulo, Brazil; 2 Center of Mathematics, Computation and Cognition, Universidade Federal do ABC, São Bernardo do Campo, São Paulo, Brazil; 3 Inter-institutional Grad Program on Bioinformatics, IME-USP, Universidade de São Paulo (USP), São Paulo, Brazil; Universitat Regensburg, GERMANY

## Abstract

Saccades were assessed in 21 adults (age 24 years, SD = 4) and 15 children (age 11 years, SD = 1), using combined functional magnetic resonance imaging (fMRI) and eye-tracking. Subjects visually tracked a point on a horizontal line in four conditions: time and position predictable task (PRED), position predictable (pPRED), time predictable (tPRED) and visually guided saccades (SAC). Both groups in the PRED but not in pPRED, tPRED and SAC produced predictive saccades with latency below 80 ms. In task versus group comparisons, children’s showed less efficient learning compared to adults for predictive saccades (adults = 48%, children = 34%, p = 0.05). In adults brain activation was found in the frontal and occipital regions in the PRED, in the intraparietal sulcus in pPRED and in the frontal eye field, posterior intraparietal sulcus and medial regions in the tPRED task. Group–task interaction was found in the supplementary eye field and visual cortex in the PRED task, and the frontal cortex including the right frontal eye field and left frontal pole, in the pPRED condition. These results indicate that, the basic visuomotor circuitry is present in both adults and children, but fine-tuning of the activation according to the task temporal and spatial demand mature late in child development.

## Introduction

Our ability to learn new motor behaviors benefits largely from observing repetitive patterns and adjusting the motor system to predict the desired motor outcome. The term prediction refers here to the ability to combine information about what was expected to happen in the motor domain with what actually did happen, as reported to the brain by the sensory system [1]. Early in our life we learn that most of the movements in our environment are rather predictable, and so prediction development allow us to reduce the processing delays and errors of tracking visual targets. Prediction can be studied with a special type of short latency saccade, the predictive saccade, produced by square-wave tracking of a visual stimulus alternating at a constant pace between fixed left and right positions. Saccades are rapid eye movements that can be generated as a reflexive response to sudden displacement of an object in the scene or voluntarily toward the position of interest [2]. Developmental studies of saccades have shown that some saccadic parameters, such as velocity and saccadic adaptation, develop throughout early childhood [3–6] while others, i.e., latency and precision, show a specific developmental curve and stabilize only in early adulthood [7–10]. Furthermore, saccadic characteristics may vary both in children and adults according to the experimental properties, i.e., saccadic latencies and errors are typically lower in visually guided saccades than in voluntary anti-saccades, in which the eye must be directed to the opposite side from the target [11–13]. Saccades have been also studied in specific developmental impairments, such as developmental dyslexia [14, 15] and attention deficit [16]. In highly functioning autism, neurofunctional impairments can be indicated by altered patterns of visual tracking and prediction, among other alterations [17]. However, until now only one study has investigated predictive saccades through square-wave tracking in normally developing children [18].

Studies using predictive saccades have reported a quick shift from visually guided to internally guided behavior in adults. As soon as subjects implicitly perceive time and position regularity in the target motion they reduce their saccadic latency to zero or to negative values [19]. When testing predictive tracking with stimulus frequencies ranging from 0.25 to 1.25 Hz, both children and adults showed reduced saccadic latency, but only in the middle range of frequencies. For frequencies that produced predictive tracking, the saccadic latency variability was similar for both groups, but children’s latencies were longer than those of adults. Adults achieved almost perfect synchrony with the target’s movement, however children’s predictive saccades were near express saccade latency, around 70 ms. Whether this indicates a slowing of the ocular motor system or rather qualitative differences in the capacity of children to predict is not clear [18]. Also, it is still to be answered if predictive tracking is supported by common saccadic circuitry in both children and adults.

Combining simultaneous eye tracking and functional magnetic resonance (fMRI) is possible to have a better insight into the developmental changes of specific behaviors as well as their underlying neural maturation process. In an anti-saccades task, for example, combined eye-tracking and fMRI study have found changes in patterns of activation and reorganization of cortical connections along with development. From childhood to adulthood, the activity of the neural system involved in inhibition decreased, while activity of neural regions involved in cognitive control of the task execution increased [20]. Moreover, functional prefrontal connectivity with other cortical and subcortical regions increased by adolescence and, furthermore, strengthened by adulthood [21].

In this study, we aimed to test predictive tracking in children and adults using a modified version of the square-wave tracking task. In this procedure, the prediction was modulated by changing the stimulus time and space predictability [22]. The purpose of our study was threefold. The first one was to identify the neural circuitry of healthy adults associated to the processing of eye-tracking targets with varying temporal and spatial predictability [22, 23]. The second one was to compare the neurofunctional patterns of activation of normally developing children with those of adults in conditions with varying temporal and spatial predictability. The last one, on a behavioral level, was to compare the saccadic latency patterns of children and adults in varying temporal and spatial predictability [18]. To our knowledge, this is the first study that collected simultaneous eye tracking and fMRI data for predictive tracking of visual target in children and adults using the same experimental set-up. We believe that it is essential to fill the gap in the literature on predictive tracking in children prior to its use in specific pediatric groups.

## Materials and methods

### Subjects

A total of 36 right-handed subjects (21 adults and 15 children) participated in the study. The adults (11 males), with a mean age of 24 (SD = 4) years, were recruited among graduate and undergraduate students. Children (9 males) with a mean age of 11 (SD = 1) years were recruited from one private basic school in São Paulo. Subjects reported no history of psychiatric or neurological illnesses, all were right-handed (according to the Edinburgh handedness inventory; [24], and had normal or corrected vision. The Ethics Committee of the Hospital das Clínicas of the Medicine Faculty, University of São Paulo approved the study under the number 0279/08. All participants and/or parents signed the written informed consent prior to the beginning of the study. Before signing the consent, the researcher in charge (author KL) explained all the procedures of the study and answered all the participants’ questions.

### Data acquisition

#### Behavioral data

**Fig 1:**
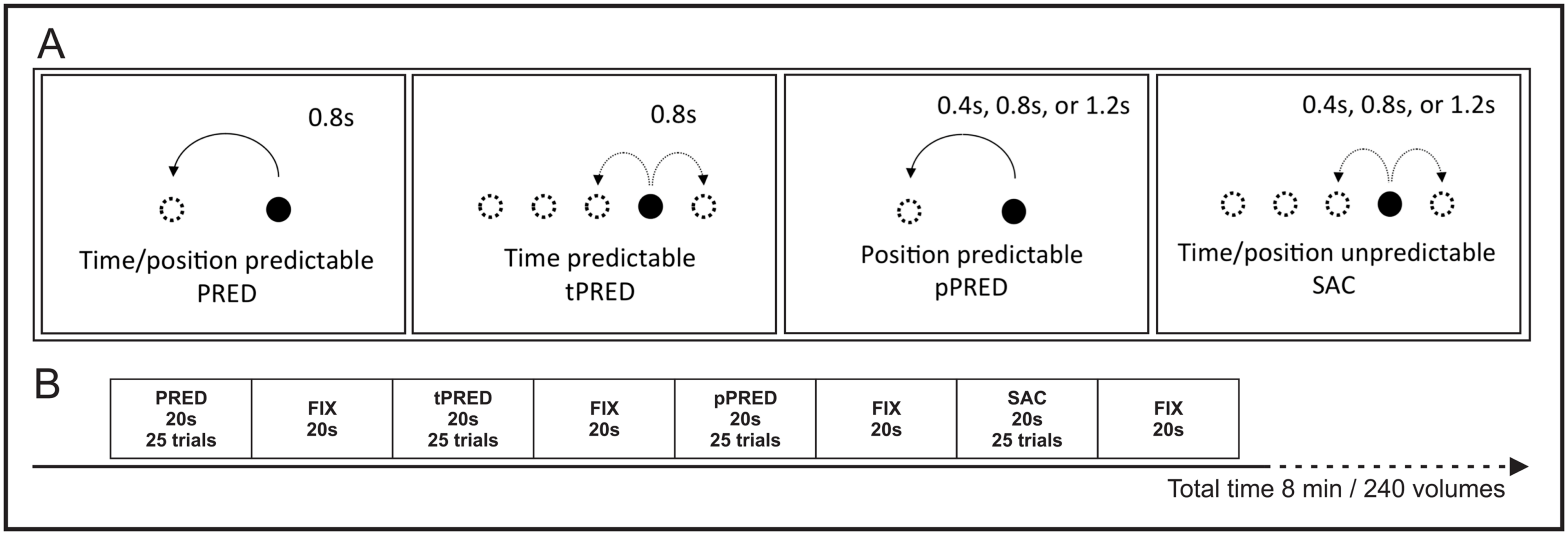
Schematic depiction of each task design (A) and experimental runs (B). (A) displays time and position specifications of trials in each task: time/position predictable (PRED), position predictable (pPRED), time predictable (tPRED), visually guided saccades (SAC). (B) shows the sequence and duration of blocks within a run. Each task was reapeated 3 times within a run in pseudo-randomized order.

Four experimental saccadic tasks were presented in a standard fMRI block design. In each task, the saccade execution was modeled by varying target predictability: mixed time/position predictable (PRED), position predictable (pPRED), time predictable (tPRED) and visually guided saccades (SAC). The task design is displayed in Fig 1. In the PRED task, the timing and position of targets was constant throughout the block. The target appeared at a constant inter-stimulus interval (ISI = 0.8 s) from left to right over 5° of visual angle (2.5° from the central baseline position). In pPRED, target position was constant as in the PRED task, with random ISIs of 0.4, 0.8 and 1.2 s. In tPRED, target timing was kept constant (ISI = 0.8 s) whereas the target could shift by 2.5° to the right or left in five different horizontal positions. The position predictability was thus decreased to 50%, except when the target was at the extreme locations on the left and right side of the screen. In order to keep the task’s overall position predictability low, the proportion of middle and extreme target positions was approximately 7: 1. In SAC, the target moved in five horizontal positions in randomly varying ISIs (0.4, 0.8, 1.2 s). To avoid giving any clue of the upcoming task, the target onset position was at the center in all conditions.

Each participant performed the task twice in two functional runs, separated by approximately 10 min of structural acquisitions. The first run was used as a training session and localizer for region of interest (ROI) analysis and the second run served for our data acquisition for the whole brain group comparisons. Each run was composed of 24 blocks of 20 s duration each. The four experimental tasks were presented three times, alternated with 12 control fixation block conditions of the same duration. The order of experimental block presentation was pseudo-randomized and was the same for all subjects. The target was a black spot (0.4° of visual angle) presented on a grey background for a total of 25 trials per block.

#### Imaging data

Imaging was performed on a 3 T MR system (Achieva, Philips Medical System, The Netherlands) with an eight-channel head receive coil. Single shot echo planar imaging (EPI), sensitivity-encoded (SENSE) for blood oxygen level-dependent (BOLD) contrast (T2*), was used with the following parameters: repetition time (TR) = 2000 ms, echo time (TE) = 30 ms, flip angle = 90°, slice thickness = 3.0 mm, gap = 0, matrix = 64 × 64, in-plane resolution 3 × 3 mm^2^. Forty slices covering the whole head were acquired with anterior commissure–posterior commissure (AC–PC) orientation in ascending order. The first four volumes were discarded to allow stabilization of magnetization. Foam padding was used to minimize head movement.

Special care was taken to ensure that children were properly acclimated to the scanner environment. All were given 15 to 20 min of training to practice the task and to keep the head stable during the fMRI procedure while listening to pre-recorded scanner noise. Additionally, a questionnaire was administered immediately after the fMRI acquisition to check the subjective impression of task difficulty, personal discomfort and general impression. The results of qualitative analyses of the reports were similar between the groups, with only one subject from each group stating ‘unwillingness to repeat the exam due to task difficulty’, few reporting high discomfort due to the position at the scanner (2 children, 1 adult), and a good general impression (most children attributed a score of 10 and adults 9).

The stimuli were projected by LCD video projector onto a screen positioned at the subjects’ feet and reflected onto the overhead mirror placed on the head coil. Eye movements were registered with an integrated MRI-compatible eye tracker (Mag Design and Engineering, sampling frequency 60Hz, precision < 1°) and processed by ViewPoint software (Arrington Research, EUA). With the subject positioned in the scanner, all subjects were equally instructed to ‘move your eyes in time with the target’ [25]. A 12-point eye tracker calibration was run immediately before the data acquisition.

Structural images were acquired in order to access white matter lesions and eventual incidental findings. The parameters were TR: 11,000 ms; TI: 2800 ms; TE: 130 ms; T = 3:18 s; matrix size: 328 × 172; FOV: 230 × 132 mm; 24 slices with 5 mm thickness, 0.5 mm inter-slice separation and 3D T1-weighted images with 1 mm isotropic voxels; TR: 7 s; TE: 3.2 s; T = 5:58s; matrix size: 240 × 240; FOV: 240 × 240 mm; flip angle = 8 and reconstruction 240. A board-certified neuroradiologist visually inspected all images.

### Data processing

#### Behavioral analysis

Behavioral data were processed offline with a custom script written in-house in Matlab^®^ (R2012a). In pre-processing, eye blinks were excluded and small drifts were corrected. Saccades were automatically identified if the eye velocity surpassed a threshold of 18° of visual angle per second, and if that velocity kept rising for a temporal window of 44 ms [26]. The first saccade within a time window of −300/+500 ms relative to the target’s appearance was considered as valid. If directed to the target, saccades were considered correct and classified according to their latency (the time between the target and saccade onset) in four categories: predictive (latency −300 to 80 ms), express (80 to 120 ms), regular (120 to 350 ms) or late (350 to 500 ms) [3, 22, 27]. If initiated out of the allowed time window or in the wrong direction, a saccade was considered an error. All detected events were visually double-checked by the experimenters (KL and RMN) to avoid misclassification.

A generalized linear mixed model with the participant as a random effect was used to assess the association between the occurrence of each type of saccade and the following fixed effects: group, task and group by task. The models were estimated using the binomial link function and an adaptive Gauss–Hermite quadrature approximation to the likelihood function [28].

To visualize whether there was an implicit learning curve in the PRED task, for each subject, the median latency (out of three) was taken for each trial within the block and then averaged for the subjects within a group. The results were graphically displayed for each group versus task. The efficiency of predictive learning, defined as a conditional probability of remaining within predictive state with saccadic latency of below 80ms [3, 27] was estimated by the following formula [29]. The probability of saccade *i* + 1 being predictive (S_*i*+1_ = P) if saccades *i* is predictive (S_*i*_ = P) is:

$$P\left(S_{i+1}=P|S_{i}=P\right)=\left(\frac{m}{M-1}\right)\left(\frac{M}{N}\right)$$

where *M* is the number of times a predictive response happened, *m* is a number of times a predictive response was followed by another predictive saccade and *N* is the total number of saccades. The predictive response is considered every saccade with latency in a range of −300 to 80ms in regard to the stimulus on-set. The conditional probability was calculated for each subject in all the PRED blocks, the data were pulled together and than the *t*-test was used to analyze the between group difference.

#### Image analysis

All functional images were processed with FSL 6.00 (Analysis Group, FMRIB, Oxford, UK; www.fmrib.ox.ac.uk). Image pre-processing of all 240 brain images (volumes) included a MCFLIRT motion correction [30] spatial smoothing using a Gaussian kernel (FWHM = 5 mm), high-pass temporal filtering (60 s) and spatial normalization to standard space (MNI 152 affine transform). Pre-processed data were analyzed using a general linear model (GLM) with FILM semi-parametric estimation of residual autocorrelation [31].

In order to assure data quality, the average motion was assessed for both groups after fMRI data processing with motion correction. The absolute head displacement during the acquisition for children was 0.47 (SD = 0.3) and adults 0.23 (SD = 0.1). There was a significant difference between groups (t-test, p < 0.01) with children having bigger head displacement than adults. However the children`s average is within the generally accepted movement range in the literature [32, 33].

The group activation maps were generated using the mixed-effects model (FLAME) [31]. The statistics images were thresholded using a cluster determined by a Z-score > 1.96 and voxel p < 0.05 in group-level analyses [30]. In order to determine activation related to predictability, group activation maps were generated for PRED, pPRED and tPRED and compared to the unpredictable SAC task. The group versus task interaction was also tested.

The peak activation foci were labeled by Juelich histological brain and Talairach Deamon FSL atlases and were visually confirmed by a board-certified neuroradiologist. In order to visualize the signal change in the ROI, a sphere of 4 mm was positioned around the coordinates of local maxima of the task × group interaction contrasts in RUN 1. For signal extraction, we used the baseline contrasts in RUN2, task (PRED, pPRED and tPRED) × baseline (cross-fixation) and the signal time series were averaged for both groups.

## Results

### Behavioral results

**Fig 2:**
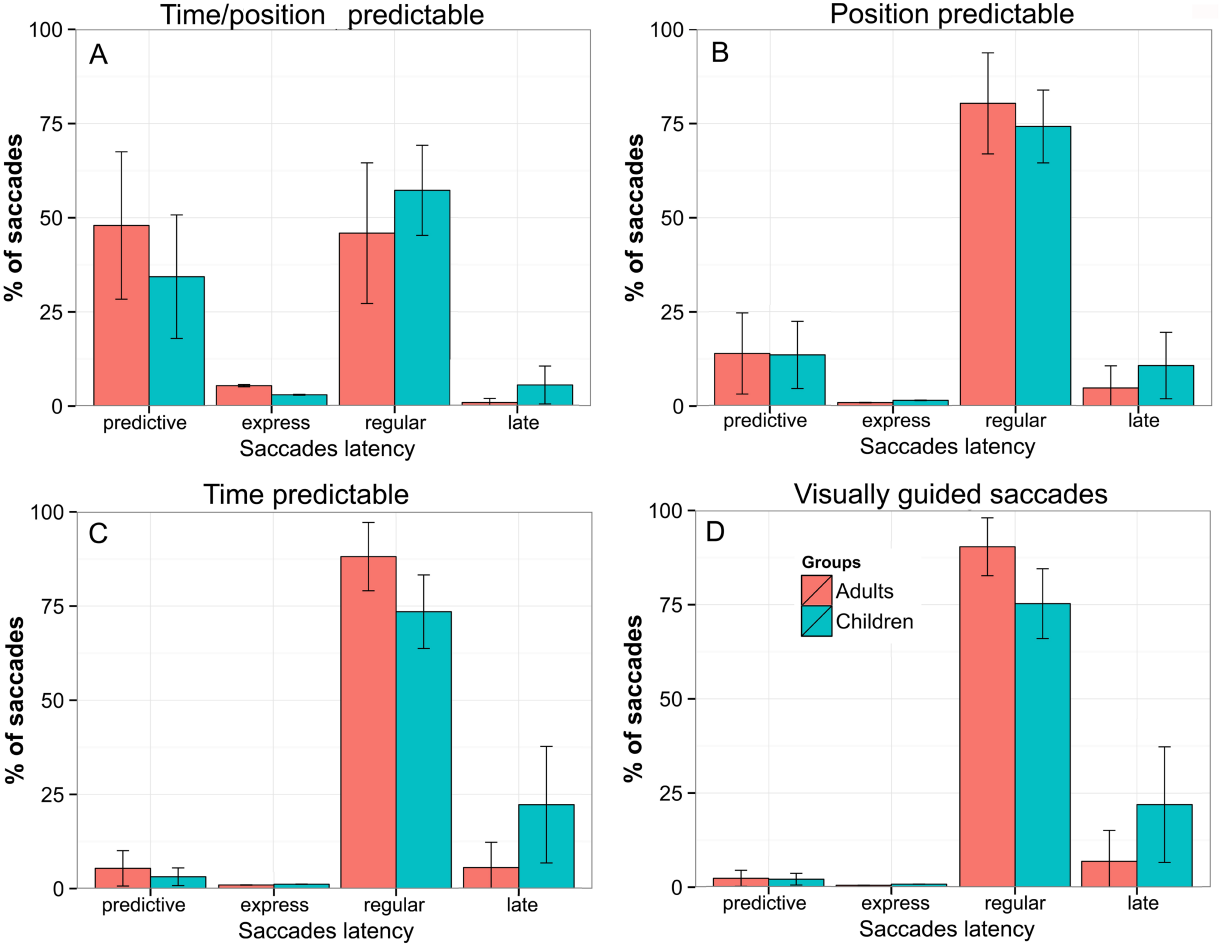
The saccades distribution for each task. The task dependent change in ocular motor pattern is displayed as the proportion of saccades (graph y-axis) generated the latency groups: predictive (-300 to 80ms), express (80 to 120ms), regular (120 to 350ms) or late saccades (350 to 500ms). The proportion of saccadets is displayed for each task and group (children and adults): (A) PRED, (B) pPRED, (C) tPRED and (D) SAC. The error bars represent the Standard Deviation.

The effect of advance knowledge of position and time on saccade latency was assessed in each task: PRED (Time/position predictable), pPRED (Position predictable), tPRED (Time predictable) and SAC (Visually guided saccades). The effect was measured by the proportion of saccades in each latency groups: predictive (latency −300 to 80 ms), express (80 to 120 ms), regular (120 to 350 ms) or late (350 to 500 ms). Overall, there was a decrease in the rate of predictive saccades along the tasks, and an increase in the rate of regular saccades in both groups from the PRED (Time/position predictable) task to pPRED (p < 0.0001) and pPRED task to tPRED (p < 0.05). Considering late saccades, the PRED task showed the lowest proportion of late saccades among tasks in both groups (p < 0.0001). The saccade distribution for each task is shown in Fig 2.

#### Latency in PRED × SAC

In the PRED task, predictive and express saccades were more frequent than in the SAC task for both groups (p < 0.0001). In addition, children made fewer predictive saccades than adults, as evidenced by a group × task interaction (model estimate = −0.583, SDE = 0.314, z = −1.856, p = 0.05, adults = 48%, children = 34%). Children also showed fewer express saccades than adults (model estimate = −1.156, SDE = 0.600, z = −1.925, p = 0.05, adults = 5%, children = 3%). On the other hand, children had a higher rate of regular saccades than adults (estimate = 1.772, SDE = 0.156, z = 11.391, p < 0.0001, adults = 46%, children = 57%). There was a marginal interaction for late saccades group × task (p < 0.05).

#### Latency in pPRED × SAC

In the pPRED task, regular and predictive latency saccades differed in frequency from the SAC task (p < 0.001). The group × task interaction was found for regular saccades (estimate = 0.842, SDE = 0.165, z = 4.763, p < 0.0001, adults = 80%, children = 74%). In pPRED, late saccades showed an effect for task and group × task (p < 0.01) with children making more late saccades than adults (adults = 4%, children = 10%, p < 0.01).

#### Latency in tPRED × SAC

In the tPRED task, the overall rate of predictive saccades was low, but they were more frequent than in the SAC task (p < 0.001), which indicates that subjects benefited from the time cue of the target. For both groups, saccades were mainly classified as regular and no statistical difference was found (adults = 88%, children = 74%).

**Fig 3:**
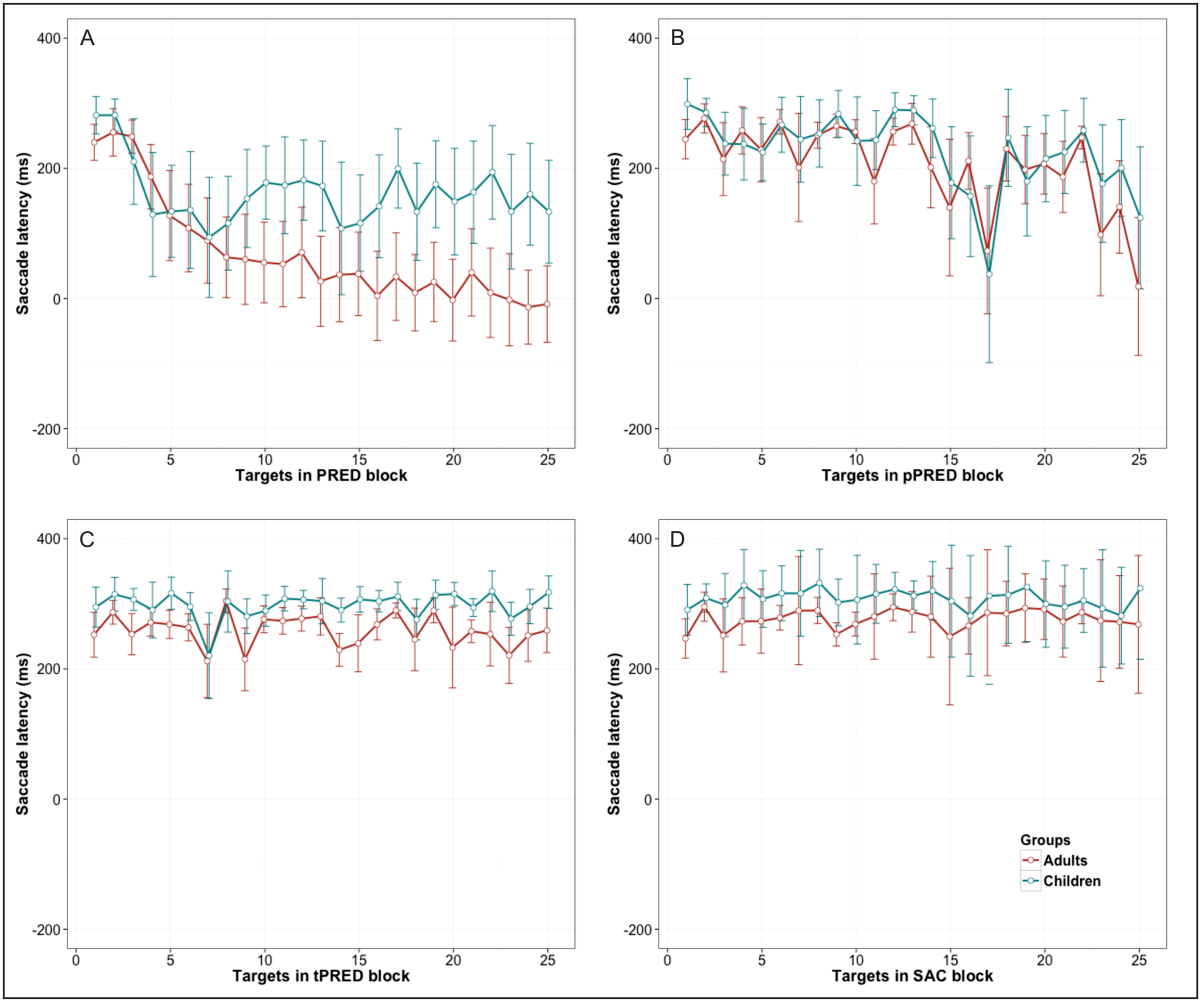
The saccadic latency along the task execution. Saccades latency for each stimuli within the block of tasks (A) PRED, (B) pPRED, (C) tPRED and (D) SAC. Median subjects’ latency for each stimuli along the task was averaged for within the group for children (red line) and adults (green line). Only in PRED task (A) saccadic latency reduced throughout the block near to 0 which indicates the learning of prediction in adults. The error bars depict the Standard Error.

In order to analyze learning effects during the task execution, the saccadic latency within the block was plotted for each stimulus and task (Fig 3). Latency was reduced throughout the block in PRED and pPRED but not in the tPRED and SAC tasks. While adults continually reduced the latency until the end of the 20 s block, children reduced the latency to approximately 150 ms, below which no further learning was evident. The learning curves for each task are depicted in Fig 3. The conditional probability of remaining within the predictive state along the task execution was higher in adults then in children (*M*_adults_ = 0.36, *SD* = 0.24; *M*_children_ = 0.21, *SD* = 0.2; *t*_(86)_ = 2.98, *p* = 0.004). In other word, this indicates that adults were more successful in maintaining a sequence of predictive saccades then children.

## fMRI results

In order to isolate areas activated by the predictability components, PRED, pPRED and tPRED were contrasted to the SAC task. Initially, whole brain exploratory analyses for adults and children are described, followed by the group *versus* task interaction. The MNI coordinates of all activated clusters are described in the Supporting Information (S1 and S2 Tables).

### Adults

**Fig 4:**
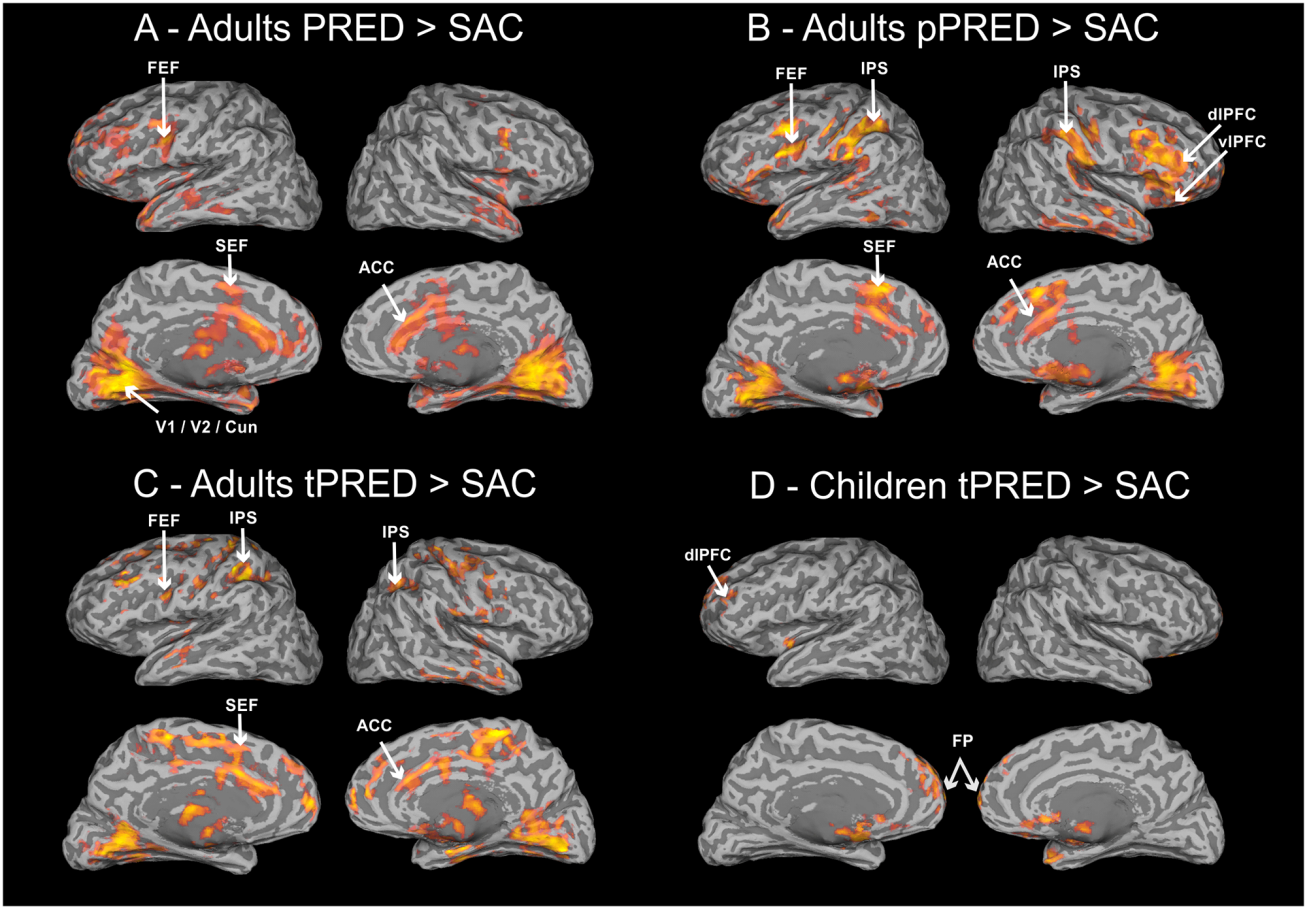
Group activations for tasks with predictable components compared with visually guided saccades. The activation pattern changed according to the tasks predictability. (A) Time/position predictable (PRED) was characterized by an increase in precentral sulcus/superior frontal sulcus and medial frontal lobe. (B) Position predictable (pPRED) the fronto-parietal and anterior cingulate cortex activation was most evident. (C) Time predictable (tPRED) activation was localized mainly in intraparietal sulcus and medial portions of the brain. (D) In children, only time predictable (tPRED) showed differential activation from SAC in frontal cortex. Statistical images were thresholded using cluster determined by Z-score > 1.96 and (corrected) cluster significance threshold of p < 0.05. The MNI coordinates of all activated clusters are described in Supplementary material (S1 Table). (Acronyms: FEF–frontal eye field; SEF—supplementary eye field; ACC–anterior cingulate cortex; IPS—intraparietal sulcus; dlPFC—dorsolateral prefrontal cortex; vlPFC—ventrolateral prefrontal cortex; FP–frontal pole).

In adults, common activation in all tasks with predictability (PRED, pPRED, tPRED) × SAC task contrast was found in the bilateral occipital cortex (V1 to V3) and dorsomedial frontal lobe corresponding to the supplementary eye field (SEF) proper. The activated regions are described below and depicted in Fig 4.

#### Activation in PRED × SAC task

The contrast of time/position predictable × unpredictable task showed activation in the frontal lobe in the SEF and frontal eye field (FEF) regions, with extended left hemisphere activation into the middle and inferior frontal gyri. On the midline, activation was found in the anterior and posterior part of the cingulate gyrus and cuneus. Subcortically, the bilateral thalamus and left putamen were activated, together with the bilateral hippocampus subiculum. Additionally, cerebellum activation was found mainly in the vermis VI, anterior lobe VI and Crus I.

#### Activation in pPRED × SAC task

For the contrast of position predictable × unpredictable task, activation in the primary visual cortex extended into the lateral occipital cortex, corresponding to V5. Frontoparietal activation was seen in the bilateral FEF, SEF and pre-SEF, extending further into the bilateral middle and inferior frontal gyri. In the parietal cortex, there was activation mainly along the anterior and middle intraparietal sulcus (IPS). The bilateral supramarginal and angular gyrus were also activated. In the temporal cortex, activation was located in the superior border of the middle temporal gyrus, and subcortically, as in the previous contrasts, the activation was found in the thalamus, putamen and cerebellum.

#### Activation in tPRED × SAC task

For the contrast of time predictable × unpredictable task, parietal activation was reduced to the bilateral posterior intraparietal sulcus, precuneus and bilateral foci in the post-central sulcus. Frontal activation was located in regions corresponding to the SEF and pre-SEF. On the midline, activation was found in the anterior and posterior parts of the cingulate gyrus and bilateral thalamus. The right middle temporal gyrus and hippocampus were also activated. In addition, cerebellum activation was found mainly in the left vermis VI, anterior lobe VI and Crus I.

### Children

No difference in activation was found in children for the PRED × SAC and pPRED × SAC contrasts. The activated regions are depicted in Fig 4.

#### Activation in tPRED × SAC

Activation was scattered mainly over the frontal cortex, located in the right frontal pole and bilateral middle frontal gyrus. Subcortically, there was activation in the bilateral nuclei caudate and only the right putamen and left thalamus.

#### Task × group interaction

**Fig 5:**
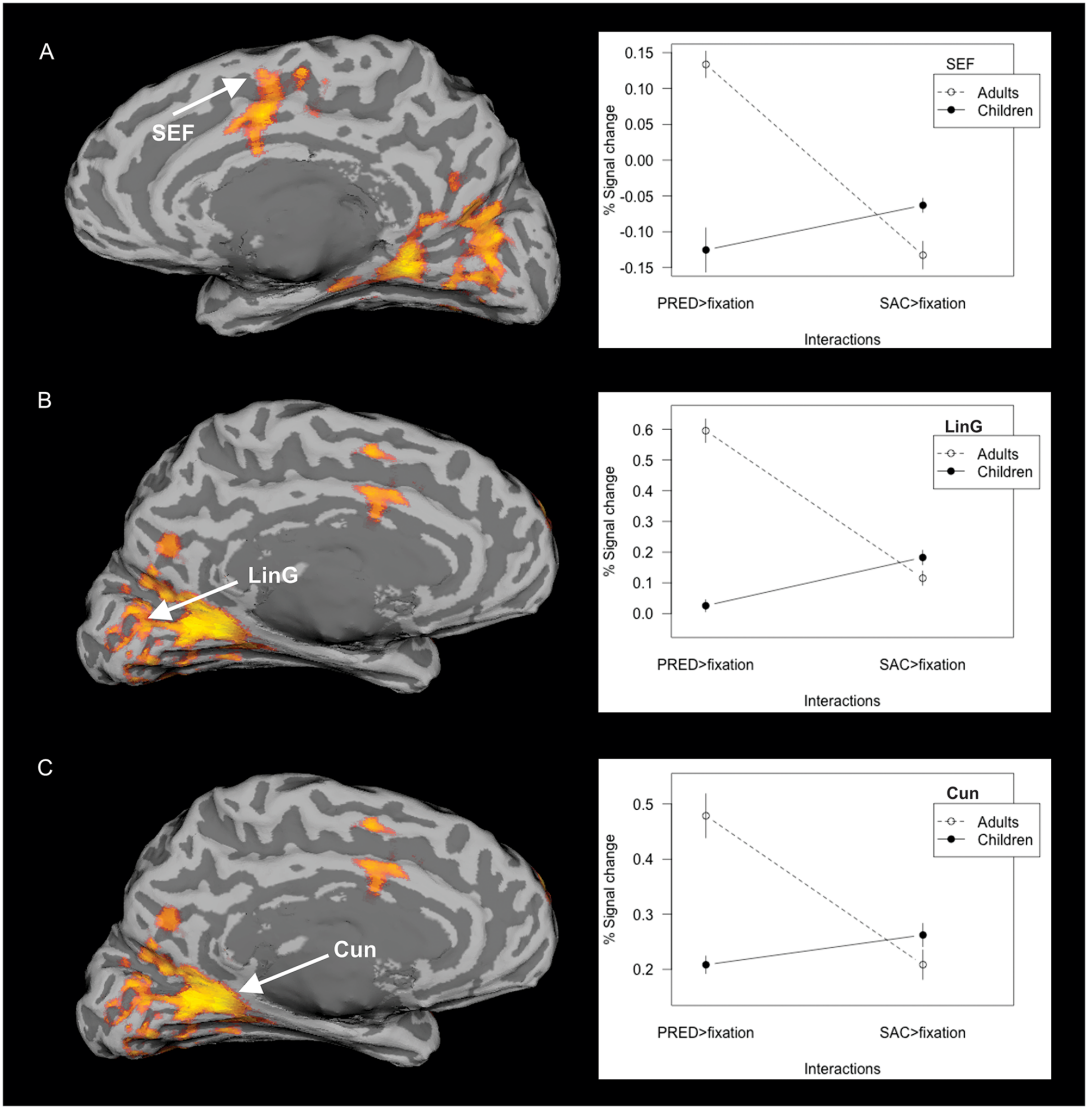
Activated regions in task × group interaction and the average signal variation. The between-group interaction contrast (on the left) in PRED × SAC task with activation in (A) frontal eye field, (B) lingual gyrus, (C) cuneus. Statistical images were thresholded using cluster determined by Z-score > 1.96 and corrected cluster significance threshold of p < 0.05. The average signal change (on the right) plotted for groups of adults and children. (Acronyms: FEF–frontal eye field; LinG–lingual gyrus; Cun–cuneus).

**Fig 6:**
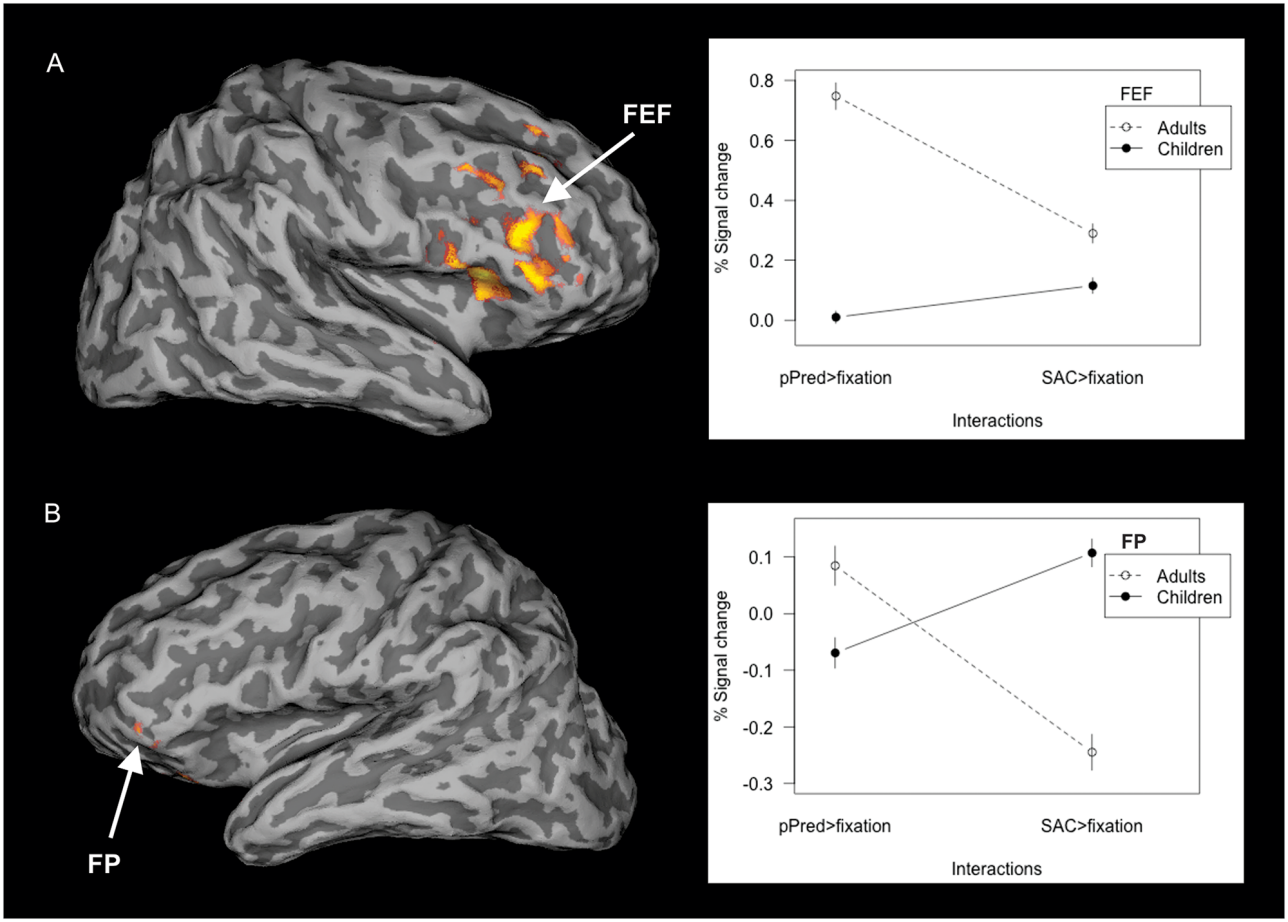
Activated regions in task × group interaction and the average signal variation. The between-group interaction contrast (on the left) in pPRED × SAC activation in (A) supplementary eye field, (B) frontal pole. Statistical images were thresholded using cluster determined by Z-score > 1.96 and corrected cluster significance threshold of p < 0.05. On the right: the average signal change plotted for groups of adults and children. Regions of interest (ROIs) were generated in three local maxima of between-groups interaction contrast (on the left. These ROIs were then used to extract the signal variation from the contrasts of task × baseline fixation in each group. (Acronyms: SEF–supplementary eye field; FP–frontal pole).

Activation was found only for adults × children in the contrasts of PRED × SAC and pPRED × SAC tasks. The activated regions and the tendency in signal change are described in Figs 5 and 6.

#### Activation in PRED × SAC task

Compared to children, adults activated more bilaterally, in primary visual areas in the lingual gyrus and cuneus. Additional activation was found in the SEF, right pre-central gyrus posterior to the FEF and left inferior frontal gyrus extending into the frontal pole. The superior board of the bilateral middle temporal gyrus was also more activated in adults than in children (Fig 5).

#### Activation in pPRED × SAC task

Activation was found to be greater in adults than in children in the lateral portion of the right FEF and right pre-central gyrus posterior to the FEF. Also, the left inferior frontal gyrus extending into frontal pole was more activated in adults than in children (Fig 6).

#### Task × group ROI signal change variation

Using the ROI peak activation expanded the whole brain analyses. The BOLD percent signal was extracted from the main ROIs (left hemisphere) from the individual contrasts of task × baseline fixation and we performed ANOVA multivariate test on the 2 groups of subjects. The tasks (PRED, pPRED, tPRED, SAC) were used as within subject and groups (children, adults) as between subjects factor.

The result showed a significant main effect for Task x Group interaction, with the percent signal change increased in adults within FEF (F_[3,78]_ = 2.74, p = 0.05) and IPS (F_[3,78]_ = 2.88, p = 0.04), but not within SEF, putamen and anterior part of the cingulate gyrus. For the task effect, Post Hoc test showed greater activation for pPRED compared to the other tasks in FEF, SEF and putamen. The posterior intraparietal sulcus showed greater activation in tPRED compared to all the other tasks. In anterior cingulate gyrus, decrease in signal differentiates the SAC task from the other tasks. The results for the task effect are depicted in Table 1.

**Table 1:** The ANOVA comparison of the signal from the main region of interest (ROI). The task × baseline mean signal variation (Std.Error) with the task as a within subject factor and *p*-value.

ROI	Mean	Mean	Mean	Mean	*F*_[3,78]_	*P*
	PRED	pPRED	tPRED	SAC		
FEF	0.42	0.66	0.47	0.36	10.61	pPRED—PRED***
	(0.06)	(0.08)	(0.07)	(0.06)		pPRED—SAC***
SEF	0.35	0.52	0.40	0.31	13.49	pPRED—PRED***
	(0.05)	(0.07)	(0.06)	(0.05)		pPRED—tPRED**
						pPRED—SAC***
IPS	0.35	0.53	0.79	0.17	19.02	tPRED—PRED***
	(0.08)	(0.10)	(0.12)	(0.05)		tPRED—pPRED*
Put.	0.25	0.29	0.24	0.16	3.19	pPRED—SAC*
	(0.04)	(0.04)	(0.04)	(0,04)		
ACC	0.03	0.05	-0.05	-0.19	9.81	SAC—PRED**
	(0.03)	(0.03)	(0.4)	(0.05)		SAC—pPRED**
						SAC—tPRED*

* < 0.05;

** < 0.01;

*** < 0.001

## Discussion

In this study, we showed that adults had an advantage from advance timing/position knowledge since they were able to reduce saccadic latency along the task execution more efficiently than children, which indicates better prediction learning. In adults, the pattern of brain activation within the ocular motor circuitry changed according to the kind of advance knowledge used in each experimental task with time only, position only or time/position cues. On the other hand, children showed small improvement in saccadic latency for predictive saccades, and the activated neural structures were mostly similar to the visually guided saccade activation. To our knowledge, there has been only one study that investigated predictive saccades behavior in children and our findings further extend the knowledge on this topic since the early studies [18].

The predictive task imposes implicit learning because of the time and positional regularities of the stimuli. To better understand the influence of timing and position cues on saccade generation within the incidental learning task, the degree of predictability was manipulated in each task. Latency decreased to a greater extent when position rather than timing was predictable, and this was more evident in adults than in children. For the advance timing knowledge, we did not find the same effect and the pattern was very similar to the visually guided saccades. This is in line with other studies with adults that have shown that position predictability leads to saccadic latency reduction by elevating the readiness of ocular motor system for the saccade execution [34–37] tested implicit learning with time structure, ordinal structure and both associated in a finger tapping task and came to similar results. Adults reduced reaction time when knowing in advance the finger’s movement order and in a mixed task but not when only ‘when to move’ was known in advance. Our results suggest that, also in the ocular motor domain, internal timing representation is formed in association with positional mapping and not as an independent temporal template. Thus, the learning is most evident when both position and time information are predictable.

According to studies in adults, the successful execution of predictive saccades is supported by an internal estimation of the stimulus timing together with the feedback of previous inter-saccade intervals, timing errors and the sequence of past movements over which this feedback is acquired. Adults are able to adjust their saccadic system to the stimulus movement after three to four saccades and move in line with the target with saccadic latencies below 80 ms [29, 38]. We found rapid adaptation to the stimulus pace in adults that showed a reduction in latency after the fourth stimulus, and a similar pattern was observed for children. The difference between the two groups was in the further synchronization of eye movements with the stimulus; while adults kept on getting more in line with the stimulus frequency, the children reached the floor effect and showed no further improvement, reaching a saccadic latency of approximately 125 ms. Our results confirm the findings of other study that tested children’s prediction for a range of target frequencies [18]. In a frequency similar to the one used in our procedure (1.25 Hz), children generated saccades with 150 ms latency. Thus, the behavioral results indicate that pre-adolescent children reduce the saccadic latency in predictive condition, but they are still not able to sustain predictive tracking (below 80ms) synchronized with the targets.

We looked into the possibility of express saccades being generated in place of predictive tracking but did not find a high rate of express saccades in children compared to adults. In fact, express saccades have been frequently reported as more frequent in children below 12 years old in pro-saccade tasks with a time gap between the central cross offset and stimuli onset [11, 39]. Klein & Foerster [11] showed that express saccade inhibition starts to develop after the age of 10–11 years and has a similar developmental course to error pro-saccades in the anti-saccade task. However, in the present task the express saccades were mostly incidental.

The behavioral differences for predictive tasks were further sustained by the neurofunctional results. We reported differences in activation pattern when comparing adults and children in tasks allowing prediction based on time/position and position information. Adults activated more than children in regions of the frontal cortex, such as the FEF and SEF, known to be responsible for visuospatial monitoring of incoming saccades [40]. Additionally, Connolly, Goodale, Menon, & Munoz [41] showed that the FEF but not the intraparietal sulcus (IPS) is the main region involved in coding the readiness and expectation to perform saccade. Visual spatiotemporal expectation has already been reported in 4 to 12 month-old infants with specific event-related potential (ERP) signals in FEF regions, indicating that at an early age frontal visual fields are involved to some extent in saccade control and visual attention [42–44]. The data from our study indicate that the neurofunctional pattern in children does not change with varying spatiotemporal expectancy when monitoring the incoming saccades. Yet, time predictable task alone led to activity localized in the frontal medial cortex and basal ganglia, possibly due to the need to compensate for poor implicit timing monitoring. This argument is supported by behavioral results showing an increase in late saccades in this task without an increase in error. The evidence indicates that the activation in the frontal cortex during anti-saccades compensates for the late maturation of secondary cortical areas [20].

In adults, the canonical ocular motor regions were activated in all tasks when contrasted against the baseline cross-fixation. Manipulation of the degree of predictability produced specific activation patterns in adults. For advance time/position knowledge, adults’ activation was similar to the results presented by [23] on predictive tracking. A large amount of activation was reported in the frontal cortex and in the anterior and posterior parts of the cingulate gyrus, putamen, thalamus and hippocampus. This study reported activation in the angular and marginal gyri that were absent in our study. We also found partially coherent results in the cerebellum, with similar activation in the vermis VI and hemispheres lobe VI, but not Crus II. For position and time tasks, our findings are divergent from a similar study by Gagnon [22], that reported activation in the dorsal FEF and caudate for position predictability and dorsal FEF, SEF, caudate and putamen activation for time predictability. We suggest that these differences stem from the use of five stimuli positions and faster pacing in our study instead of three positions and longer ISIs used by Gagnon. We showed activation along the intraparietal sulcus together with the frontal FEF/SEF regions for the position task, while in the time task, frontal region activation was restricted to the SEF, posterior intraparietal sulcus and precuneus. Studies in monkeys have largely reported on spatial tuning of the lateral intraparietal cortex (LIP), but have only recently shown a specific role of the SEF and the neurons within this area in sequential state representation and timing [45]. In other words, intraparietal regions code the direction of upcoming saccade with the SEF providing detailed information on upcoming motor intention and the timing of the saccade sequence [46]. Transposing this model to human prediction, it would explain the change in activation pattern we found in the time and position tasks, with larger parietal activation for position monitoring and more evident frontal activation for time and time/position monitoring. The main implication of our study is the absence of this activation pattern in pre-adolescent children, and it should be taken into account in clinical population studies that explore predictive saccade abilities [17]. Differently from Ross & Ross [18], our data indicate that the slowing of the ocular motor system in children during predictive saccades is a behavioral trait of neurofunctional differences when compared to similar behavior in adults. Thus a special care should be taken when comparing the children’s performance in predictive saccades of healthy or clinical population to the adult literature.

This study has some limitations. fMRI acquisition in children is challenging due to the high frequency of subjects’ head movement, which was also the case in this study. We found more movement in the children group; it was, however, within normally accepted limits reported by studies in children and clinical populations [47]. In the pPRED latency plot (Fig 3), unexpected short latencies were found for stimuli 17 and 25. We believe that this could be due to the nature of the stimulus or some alias effect during synchronization between triggering the system and the programmed stimulus-onset asynchrony.

In conclusion, our results indicate that the basic visuomotor circuitry present in pre-adolescents and adults is similar. However, fine-tuning of this system according to the task’s temporal and spatial demand is not yet matured in children, probably due to the interplay of inefficient error-feedback processing, error correction and timing monitoring. Thus, we believe that an efficient predictive behavior can be found only late in child development and until it is fully ready, reflexive-like saccades are executed. The implication of this finding is important when planning child developmental fMRI studies involving eye movements (learning paradigms based on visual presentation of stimulus). Our data may also help in the choice of neuroimaging variables as end points for psychological and/or pharmacological clinical trials.

## Supporting information

S1 Table Brain regions activated in tasks with predictable components (PRED, pPRED, tPRED) compared to SAC. MNI peak voxels coordinates for adults.
(PDF)

S2 Table Brain regions activated in tasks with predictable components (PRED, pPRED, tPRED) compared to SAC. MNI peak voxels coordinates for children.
(PDF)

## References

[1] Shadmehr R, Smith MA, Krakauer JW. Error Correction, Sensory Prediction, and Adaptation in Motor Control. Annu Rev. Neurosci. 2010 doi: 10.1146/annurev-neuro-060909-153135 20367317 10.1146/annurev-neuro-060909-153135

[2] Kowler E. Eye movements: The past 25 years. Vision Research. 2011;51(13): 1457–1483. doi: 10.1016/j.visres.2010.12.014 21237189 10.1016/j.visres.2010.12.014 PMC3094591

[3] Bucci MP, Seassau M. Saccadic eye movements in children: a developmental study. Experimental Brain Research. 2012;222(1–2): 21–30. doi: 10.1007/s00221-012-3192-7 22836522 10.1007/s00221-012-3192-7

[4] Doré-Mazars K, Vergilino-Perez D, Lemoine C, Bucci MP. Adaptation of reactive saccades in normal children. Investigative Ophthalmology and Visual Science. 2011;52(7): 4813–4818. doi: 10.1167/iovs.10-6626 21421872 10.1167/iovs.10-6626

[5] Irving EL, Steinbach MJ, Lillakas L, Babu RJ, Hutchings N. Horizontal saccade dynamics across the human life span. Investigative Ophthalmology & Visual Science. 2006; 47(6): 2478–84. doi: 10.1167/iovs.05-1311 16723459 10.1167/iovs.05-1311

[6] Salman MS, Sharpe JA, Eizenman M, Lillakas L, Westall CTT, Steinbach MJ. Saccades in children. Vision Research. 2006;46(8–9): 1432–9. doi: 10.1016/j.visres.2005.06.011 16051306 10.1016/j.visres.2005.06.011

[7] Eckstein MP. Visual search: a retrospective. Journal of Vision. 2011;11(5): 1–36. doi: 10.1167/11.5.14 22209816 10.1167/11.5.14

[8] Ego C, Orban de Xivry JJ, Nassogne MC, Yüksel D, Lefèvre P. The saccadic system does not compensate for the immaturity of the smooth pursuit system during visual tracking in children. Journal of Neurophysiology. 2013;110: 358–67. doi: 10.1152/jn.00981.2012 23615545 10.1152/jn.00981.2012

[9] Gredebäck G, Ornkloo H, von Hofsten C. The development of reactive saccade latencies. Experimental Brain Research. 2006; 173(1): 159–64. doi: 10.1007/s00221-006-0376-z 16489432 10.1007/s00221-006-0376-z

[10] Luna B, Velanova K, Geier CF. Development of eye-movement control. Brain and Cognition, 2008;68(3): 293–308. doi: 10.1016/j.bandc.2008.08.019 18938009 10.1016/j.bandc.2008.08.019 PMC2731686

[11] Klein C, Foerster F. Development of prosaccade and antisaccade task performance in participants aged 6 to 26 years. Psychophysiology, 2001;38(2): 179–89. Avaliable from: http://www.ncbi.nlm.nih.gov/pubmed/11347863 11347863

[12] Ordaz S, Davis S, Luna B. Effects of response preparation on developmental improvements in inhibitory control. Acta Psychologica, 2010;134(3): 253–63. doi: 10.1016/j.actpsy.2010.02.007 20347061 10.1016/j.actpsy.2010.02.007 PMC2885497

[13] Velanova K, Wheeler ME, Luna B. The maturation of task set-related activation supports late developmental improvements in inhibitory control. The Journal of Neuroscience, 2009;29(40): 12558–67. doi: 10.1523/JNEUROSCI.1579-09.2009 19812330 10.1523/JNEUROSCI.1579-09.2009 PMC2788337

[14] Biscaldi M, Fischer B, Hartnegg K. (2000). Voluntary saccadic control in dyslexia. Perception, 29(5), 509–521. doi: 10.1068/p2666a 10992950 10.1068/p2666a

[15] Liddle E, Chou YJ, Jackson S. Perisaccadic mislocalization in dyslexia. Neuropsychologia, 2009;47(1): 77–82. doi: 10.1016/j.neuropsychologia.2008.08.013 18789347 10.1016/j.neuropsychologia.2008.08.013

[16] Klein CH, Raschke A, Brandenbusch A. Development of pro- and antisaccades in children with attention-deficit hyperactivity disorder (ADHD) and healthy controls. Psychophysiology, 2003;40(1): 17–28. Avaliable from:http://www.ncbi.nlm.nih.gov/pubmed/12756978 12756978 10.1111/1469-8986.00003

[17] Goldberg MC, Lasker AG, Zee DS, Garth E, Tien A, Landa RJ. Deficits in the initiation of eye movements in the absence of a visual target in adolescents with high functioning autism. Neuropsychologia, 2002; 40(12): 2039–2049. doi: 10.1016/S0028-3932(02)00059-3 12208001 10.1016/s0028-3932(02)00059-3

[18] Ross SM, Ross LE. Children’s and adults' predictive saccades to square-wave targets. Vision Research, 1987; 27(12): 2177–2180. doi: 10.1016/0042-6989(87)90131-3 3447365 10.1016/0042-6989(87)90131-3

[19] Shelhamer M, Joiner WM. Saccades exhibit abrupt transition between reactive and predictive; predictive saccade sequences have long-term correlations. Journal of Neurophysiology, 2003; 90(4): 2763–9. doi: 10.1152/jn.00478.2003 14534279 10.1152/jn.00478.2003

[20] Velanova K, Wheeler ME, Luna B. Maturational changes in anterior cingulate and frontoparietal recruitment support the development of error processing and inhibitory control. Cerebral Cortex, 2008;18(11): 2505–22. doi: 10.1093/cercor/bhn012 18281300 10.1093/cercor/bhn012 PMC2733315

[21] Hwang K, Velanova K, Luna B. Strengthening of top-down frontal cognitive control networks underlying the development of inhibitory control: a functional magnetic resonance imaging effective connectivity study. The Journal of Neuroscience, 2010;30(46): 15535–15545. doi: 10.1523/JNEUROSCI.2825-10.2010 21084608 10.1523/JNEUROSCI.2825-10.2010 PMC2995693

[22] Gagnon D, O’Driscoll GA, Petrides M, Pike GB. The effect of spatial and temporal information on saccades and neural activity in oculomotor structures. Brain, 2002;125: 123–39. Avaliable from: http://www.ncbi.nlm.nih.gov/pubmed/11834598 11834598 10.1093/brain/awf005

[23] Simó LS, Krisky CM, Sweeney JA. Functional neuroanatomy of anticipatory behavior: dissociation between sensory-driven and memory-driven systems. Cerebral Cortex, 2005; 15(12): 1982–91. doi: 10.1093/cercor/bhi073 15758195 10.1093/cercor/bhi073

[24] Oldfield RC. The assessment and analyses of handedness: The Edinburgh Inventory. Neuropsychology, 1979; 9(1): 97–113. Avaliable from: http://www.ncbi.nlm.nih.gov/pubmed/5146491 10.1016/0028-3932(71)90067-4 5146491

[25] Isotalo E, Lasker AG, Zee DS. Cognitive influences on predictive saccadic tracking. Experimental Brain Research, 2005;165(4): 461–9. doi: 10.1007/s00221-005-2317-7 16025290 10.1007/s00221-005-2317-7

[26] Ordaz S, Foran W, Velanova KS, Luna B. Longitudinal growth curves of brain function underlying inhibitory control through adolescence. Journal of Neuroscience, 2013;33(46): 18109–18124. doi: 10.1523/JNEUROSCI.1741-13.2013 24227721 10.1523/JNEUROSCI.1741-13.2013 PMC3828464

[27] Fischer B, Biscaldi M, Gezeck S. On the development of voluntary and reflexive components in human saccade generation. Brain Research, 1997;754(1–2): 285–97. Avaliable from: http://www.ncbi.nlm.nih.gov/pubmed/9134986 9134986 10.1016/s0006-8993(97)00094-2

[28] Bates D, Mächler M, Bolker BM, Walker SC. Fitting linear mixed-effects models using lme4. Journal of Statistical Software, 2015; Avaliable from: http://arxiv.org/abs/1406.5823

[29] Joiner WM, Shelhamer M. A model of time estimation and error feedback in predictive timing behavior. Journal of Computational Neuroscience, 2009;26(1): 119–38. doi: 10.1007/s10827-008-0102-x 18563546 10.1007/s10827-008-0102-x

[30] Worsley KJ, Liao CH, Aston J, Petre V, Duncan GH, Morales F et al A general statistical analysis for fMRI data. NeuroImage, 2002; 15(1): 1–15. doi: 10.1006/nimg.2001.0933 11771969 10.1006/nimg.2001.0933

[31] Woolrich MW, Ripley BD, Brady M, Smith SM. Temporal autocorrelation in univariate linear modeling of FMRI data. NeuroImage, 2001;14(6): 1370–86. doi: 10.1006/nimg.2001.0931 11707093 10.1006/nimg.2001.0931

[32] Emerson RW, Cantlon JF. Continuity and change in children`s longitudinal neural response to number. Dev. Sci., 2015;18(2): 314–26. doi: 10.1111/desc.12215 25051893 10.1111/desc.12215 PMC4303560

[33] Yuan W, Altaye M, Ret J, Schmithorst V, Byars AW, Plante E, et al Quantification of head motion in children during various fMRI language tasks. Human Brain Mapping, 2009;30(5): 1481–1489. doi: 10.1002/hbm.20616 18636549 10.1002/hbm.20616 PMC2763570

[34] Curtis CE, Connolly JD. Saccade preparation signals in the human frontal and parietal cortices. Journal of Neurophysiology, 2008;99(1): 133–145. doi: 10.1152/jn.00899.2007 18032565 10.1152/jn.00899.2007 PMC2671395

[35] Oswal A, Ogden M, Carpenter RHS. The time course of stimulus expectation in a saccadic decision task. Journal of Neurophysiology, 2007;97(4): 2722–2730. doi: 10.1152/jn.01238.2006 17267751 10.1152/jn.01238.2006

[36] Tark KJ, Curtis CE. Persistent neural activity in the human frontal cortex when maintaining space that is off the map. Nature Neuroscience, 2009;12(11): 1463–1468. doi: 10.1038/nn.2406 19801987 10.1038/nn.2406 PMC3171293

[37] O’Reilly JX, McCarthy KJ, Capizzi M, Nobre AC. Acquisition of the temporal and ordinal structure of movement sequences in incidental learning. Journal of Neurophysiology, 2008;99(5): 2731–5. doi: 10.1152/jn.01141.2007 18322005 10.1152/jn.01141.2007

[38] Wong AL, Shelhamer M. Similarities in error processing establish a link between saccade prediction at baseline and adaptation performance. Journal of Neurophysiology, 2014;111(10): 2084–93. doi: 10.1152/jn.00779.2013 24598520 10.1152/jn.00779.2013 PMC4044342

[39] Fischer B, Weber H. Effects of stimulus conditions on the performance of antisaccades in man. Experimental Brain Research, 1997;116(2): 191–200. doi: 10.1007/PL00005749 9348120 10.1007/pl00005749

[40] McDowell JE, Dyckman KA, Austin BP, Clementz BA. Neurophysiology and neuroanatomy of reflexive and volitional saccades: evidence from studies of humans. Brain and Cognition, 2008;68(3): 255–70. doi: 10.1016/j.bandc.2008.08.016 18835656 10.1016/j.bandc.2008.08.016 PMC2614688

[41] Connolly JD, Goodale MA, Menon RS, Munoz DP. Human fMRI evidence for the neural correlates of preparatory set. Nature Neuroscience, 2002;5(12): 1345–52. doi: 10.1038/nn969 12411958 10.1038/nn969

[42] Canfield RL, Kirkham NZ. Infant Cortical Development and the Prospective Control of Saccadic Eye Movements. Infancy, 2001;2(2): 197–211. doi: 10.1207/S15327078IN0202_5

[43] Rose SA, Feldman JF, Jankowski JJ, Caro DM. A longitudinal study of visual expectation and reaction time in the first year of life. Child Development, 2002;73(1): 47–61. Avaliable from: http://www.ncbi.nlm.nih.gov/pubmed/14717243 14717243 10.1111/1467-8624.00391

[44] Wentworth N, Haith MM. Infants’ acquisition of spatiotemporal expectations. Developmental Psychology, 1998;34(2): 247–257. doi: 10.1037/0012-1649.34.2.247 9541777 10.1037//0012-1649.34.2.247

[45] Campos M, Breznen B, Andersen RA. A neural representation of sequential states within an instructed task. Journal of Neurophysiology, 2010;104(5): 2831–2849. doi: 10.1152/jn.01124.2009 20739594 10.1152/jn.01124.2009 PMC2997039

[46] Campos M, Breznen B, Andersen RA. Separate Representations of Target and Timing Cue Locations in the Supplementary Eye Fields. J Neurophysiol, 2009; 101(1): 448–459. doi: 10.1152/jn.90704.2008 19005001 10.1152/jn.90704.2008 PMC3815215

[47] Church JA, Petersen SE, Schlaggar BL. The “Task B problem” and other considerations in developmental functional neuroimaging. Human Brain Mapping, 2010;31(6): 852–62. doi: 10.1002/hbm.21036 20496376 10.1002/hbm.21036 PMC3468298

