# Supplementary material for: Predictive saccades in children and adults: A combined fMRI and eye tracking study
Source: PLoS One. 2018 May 2;13(5):e0196000. doi: 10.1371/journal.pone.0196000 (PMC5931500; doi:10.1371/journal.pone.0196000)
Supplement: S1 Table — MNI peak voxels coordinates for adults. (PDF) [file pone.0196000.s001.pdf]

S1 Table. Brain regions activated in tasks with predictable components (PRED, pPRED, tPRED) compared to SAC. MNI peak voxels coordinates for adults.

		**PRED>SAC**		**pPRED>SAC**		**tPRED>SAC**	
**Region**	**Side**	**Z-score**	**Coordinates MNI (x/y/z)**	**Z-score**	**Coordinates MNI (x/y/z)**	**Z-score**	**Coordinates MNI (x/y/z)**
OCCIPITAL CORTEX							
Lingual Gr /V1	L	5.55	-8/86/0	5.04	-10/-88/-2	4.24	0/-90/-8
Lingual Gr /V1	R	5.02	8/-72/-6	3.50	10/-76/4	3.6	10/-78/2
Lingual Gr/V2	L	5.04	-14/-74/2	3.80	-10/78/-2	3.78	-12/-72/0
Lingual Gr/V2	R	4.97	12/-82/4	3.60	12/-76/-2	3.75	14/-80/2
Cuneus	L/R	4.59	-6/-78/10	---	---	---	---
Lateral Occipital Cortex/V5	L	---	---	3.17	-58/-64/-2	---	---
Lateral Occipital Cortex/V5	R	---	---	4.15	2/-88/12	---	---
PARIETAL CORTEX							
Ant. intraparietal sulco	L	---	---	4.83	-58/-50/40	---	---
Ant. intraparietal sulco	R	---	---	3.36	52/-40/48	---	---
Middle intraparietal sulco	L	---	---	4.31	-42/-58/48	4.3	-44/-58/54
Middle intraparietal sulco	R	---	---	3.39	50/-56/48	---	---
Posterior intraparietal sulco	L	---	---	---	---	2.83	-22/66/50
Posterior intraparietal sulco	R	---	---	---	---	2.5	26/-74/50
Supramarginal gyrus	L	---	---	4.83	-58/-50/40	---	---
Supramarginal gyrus	R	---	---	3.60	56/-42/32	---	---
Angular gyrus	L	---	---	2.89	-56/-58/12	---	---
Angular gyrus	R	---	---	---	---	---	---
Precuneus	L/R	---	---	---	---	4.37	4/-54/66
Pos-central/ anterior intraparietal sulco	L	---	---	---	---	3.8	-44/-34/34
Pos-central/ anterior intraparietal sulco	R	---	---	---	---	2.66	36/-34/38
FRONTAL CORTEX							
Dorsomedial frontal lobe/SEF proper	L/R	3.62	8/-18/54	3.25	2/-2/62	3.32	0/-6/50
Dorsomedial frontal lobe/pré-SEF	L/R	---	---	2.80	6/24/42	2.7	6/26/52
Precentral sulcus/FEF	L	3.69	-40/-18/38	4.71	-46/-12/44	---	---
Precentral sulcus/FEF	R	3.11	40/16/38	4.58	44/2/28	---	---
Inferior frontal gyrus (posterior part)	L	3.70	-46/6/-6	3.62	-46/12/-2	---	---
Inferior frontal gyrus (Posterior part)	R	---	---	3.2	54/6/12	---	---
Inferior fr. gyrus/ vlPFC	L	3.28	-46/40/0	3.4	-46/44/-2	---	---

Inferior fr. gyrus/ vlPFC	R	---	---	3.2	46/44/-8	---	---
Middle frontal gyrus/ dlPFC	L	3.80	-32/48/22	3.02	-30/50/22	---	---
Middle frontal gyrus/ dlPFC	R	---	---	3.68	24/50/22	---	---
Middle frontal gyrus (posterior part)	L	3.90	-54/28/14	---	---	4.06	-40/14/52
Middle frontal gyrus (posterior part)	R	---	---	2.92	42/38/10	---	---
TEMPORAL							
Middle temporal gyrus	L	---	---	---	---	---	---
Middle temporal gyrus	R	---	---	3.49	50/-28/-6	3.9	50/-28/-6
Hipocampus subiculum	L	2.33	-30/-32/-10	---	---	---	---
Hipocampus subiculum	R	3.20	20/-30/-8	---	---	3.2	34/-32/-14
OTHER REGIONS							
Anterior Cingulate gyrus	L/R	4.55	0/6/40	---	---	3.09	2/8/40
Posterior Cingulate gyrus	L/R	3.80	0/-28/24	---	---	3.32	2/-36/26
Putamen	L	3.20	-22/8/6	3.5	-20/6/-4	---	---
Putamen	R	---	---	3.6	20/6/0	---	---
Thalamus	L	2.90	10/-4/12	3.6	-8/-14/4	3.5	-14/-26/6
Thalamus	R	2.90	8/-6/14	3.8	12/-14/6	---	---
Cerebelum Vermis VI	L	2.90	-4/-72/-26	3.42	-4/-72/-26	3.39	-2/-80/-14
Cerebelum Vermis VI	R	---	---	---	---	---	---
Cerebelum- anterior lobe VI	L	2.80	-24/-58/-24	4.12	-34/-60/-28	3.5	-32/-50/-34
Cerebelum- anterior lobe VI	R	2.91	10/-64/-22	4.45	30/-60/-26	---	---
Cerebelum- Cruz I	L	3.03	-4/-80/-20	---	---	3.5	-48/-60/-34
Cerebelum- Cruz I	R	---	---	---	---	---	---

MNI peak voxel activation (x,y,z in mm, MNI-152, highest Z values within clusters). Abbreviations from top: SEF - supplementary eye field, FEF - frontal eye field, FEF - frontal eye field; vlPFC - ventrolateral prefrontal cortex, dlPFC – dorsolateral prefrontal cortex. PRED – time/position predictable, pPRED – position predictable, tPRED – time predictable, SAC – visually guided saccades.
