# Supplementary material for: Predictive saccades in children and adults: A combined fMRI and eye tracking study
Source: PLoS One. 2018 May 2;13(5):e0196000. doi: 10.1371/journal.pone.0196000 (PMC5931500; doi:10.1371/journal.pone.0196000)
Supplement: S2 Table — MNI peak voxels coordinates for children. (PDF) [file pone.0196000.s002.pdf]

S2 Table. Brain regions activated in tasks with predictable components (PRED, pPRED, tPRED) compared to SAC. MNI peak voxels coordinates for children.

		PRED>SAC		pPRED>SAC		tPRED>SAC	
**Region**	**Side**	**Z-score**	**Coordinates MNI (x/y/z)**	**Z-score**	**Coordinates MNI (x/y/z)**	**Z-score**	**Coordinates MNI (x/y/z)**
FRONTAL CORTEX							
Frontal pole	R	4.03	18/62/26				
Middle frontal gyrus/ dlPFC	L	3.43	-8/44/30				
Middle frontal gyrus/ dlPFC	R	3.35	23/60/22				
OTHER REGIONS							
Caudate	L	3.38	-6/-4/16				
Caudate	R	3.39	12/10/14				
Putamen	L	---	---				
Putamen	R	3.22	20/6/2				
Thalamus	L	3.15	-6/-14/10				
Thalamus	R	---	---				

MNI peak voxel activation (x,y,z in mm, MNI-152, highest Z values within clusters). Abbreviations from top: dlPFC – dorsolateral prefrontal cortex. PRED – time/position predictable, pPRED – position predictable, tPRED – time predictable, SAC – visually guided saccades.
